# Correlation of omega-3 levels in serum phospholipid from 2053 human blood samples with key fatty acid ratios

**DOI:** 10.1186/1475-2891-8-58

**Published:** 2009-12-24

**Authors:** Bruce J Holub, Mike Wlodek, William Rowe, Jerry Piekarski

**Affiliations:** 1 Department of Human Health and Nutritional Sciences, University of Guelph, Guelph, Ontario, N1G 2W1, Canada; 2 Nutrasource Diagnostics Inc. 120 Research Lane, Suite 203 University of Guelph Research Park Guelph, Ontario, N1G 0B4, Canada; 3 Lipid Analytical Laboratories Inc. 150 Research Lane, Room 100 University of Guelph Research Park Guelph, Ontario, N1G 4T4, Canada

## Abstract

**Background:**

This research was conducted to explore the relationships between the levels of omega-3 fatty acids in serum phospholipid and key fatty acid ratios including potential cut-offs for risk factor assessment with respect to coronary heart disease and fatal ischemic heart disease.

**Methods:**

Blood samples (n = 2053) were obtained from free-living subjects in North America and processed for determining the levels of total fatty acids in serum phospholipid as omega-3 fatty acids including EPA (eicosapentaenoic acid, 20:5 n-3) and DHA (docosahexaenoic acid, 22:6 n-3) by combined thin-layer and gas-liquid chromatographic analyses. The omega-3 levels were correlated with selected omega-6: omega-3 ratios including AA (arachidonic acid, 20:4n-6): EPA and AA:(EPA+DHA). Based on previously-published levels of omega-3 fatty acids considered to be in a 'lower risk' category for heart disease and related fatality, 'lower risk' categories for selected fatty acid ratios were estimated.

**Results:**

Strong inverse correlations between the summed total of omega-3 fatty acids in serum phospholipid and all four ratios (omega-6:omega-3 (n-6:n-3), AA:EPA, AA:DHA, and AA:(EPA+DHA)) were found with the most potent correlation being with the omega-6:omega-3 ratio (R^2 ^= 0.96). The strongest inverse relation for the EPA+DHA levels in serum phospholipid was found with the omega-6: omega-3 ratio (R^2 ^= 0.94) followed closely by the AA:(EPA+DHA) ratio at R^2 ^= 0.88. It was estimated that 95% of the subjects would be in the 'lower risk' category for coronary heart disease (based on total omega-3 ≥ 7.2%) with omega-6:omega-3 ratios <4.5 and AA:(EPA+DHA) ratios <1.4. The corresponding ratio cut-offs for a 'lower risk' category for fatal ischemic heart disease (EPA+DHA ≥ 4.6%) were estimated at < 5.8 and < 2.1, respectively.

**Conclusions:**

Strong inverse correlations between the levels of omega-3 fatty acids in serum (or plasma) phospholipid and omega-6: omega-3 ratios are apparent based on this large database of 2053 samples. Certain fatty acid ratios may aid in cardiovascular disease-related risk assessment if/when complete profiles are not available.

## Introduction

The fatty acid composition of serum (or plasma) phospholipid has become established as a valid biochemical marker for assessing the physiological status of various fatty acids including predictive correlations with the dietary intakes of fish-derived omega-3 fatty acids including EPA (eicosapentaenoic acid, 20:5 n-3) and DHA (docosahexaenoic acid, 22:6 n-3) [1,2]. Population studies have shown an inverse relation between total omega-3 fatty acids in blood serum phospholipid and the risk for coronary heart disease with percentages of total omega-3 ≥ 7.2 being associated with a 31% lower risk [3,4]. Furthermore, DHA levels (as percent of total fatty acids in serum phospholipid) of ≥ 4.5 have been associated with a 34% lower risk for coronary heart disease [3,4]. With respect to the risk of fatal ischemic heart disease, EPA+DHA (summed) levels amounting to at least 4.6% of total fatty acids in the serum phospholipid were associated with a 70% lower risk as compared to those with much lower levels of these fatty acids [4,5].

Since the omega-6 fatty acid, AA (arachidonic acid, 20:4 n-6), found in abundance in various cells and tissues including serum phospholipid can be readily converted into pro-inflammatory eicosanoids and other products associated with inflammatory processes and chronic disorders in contrast to EPA [6], the AA:EPA ratio in serum phospholipid has been studied in relation to the risk of chronic disorders. These studies have, as an example, indicated that the AA/EPA ratio in serum (or plasma) phospholipid correlates positively with clinical symptoms of depression [7,8]; furthermore, higher ratios of AA:DHA were associated with greater neuroticism [8]. Others have implicated the abundance of the summed omega-6 relative to the omega-3 fatty acids in human plasma phospholipid with respect to chronic disorders [9]. While the percentages of omega-3 fatty acids or key ratios (e.g., AA:EPA ratio) in serum (or plasma) phospholipid and disease risk are often given in published papers, the percentages and various key fatty acid ratios are usually not provided simultaneously.

In view of the availability of an extremely large database (over 2000 samples) of subjects from North America with complete fatty acid profiles of their serum phospholipid, the following were of interest: 1) to determine the relationship between the percentage of total fatty acids in serum phospholipid as total omega-3, DHA, and (EPA+ DHA)-risk factors for coronary heart disease and fatal ischemic heart disease (plus other chronic disorders) to the various ratios (omega-6:omega-3, AA:EPA, AA:DHA, and AA:(EPA+DHA); 2) to compare the relative strengths of these correlations (fatty acid percentages with ratios); 3) to determine the corresponding cut-points (95 percentile) for the (omega-6:omega-3), AA:EPA, AA:DHA, and AA:(EPA+DHA) ratios which are associated with a 95% likelihood of the percentage of total fatty acids in serum phospholipid as omega-3, DHA, and EPA+DHA being in the aforementioned 'lower-risk' category [3-5].

## Methods

### Study Population and Blood Samples

Blood samples (n = 2053) were obtained from free-living subjects (both genders) from across Canada and the United States who initiated contact with their health professional or health care provider to request analyses of their serum phospholipid for fatty acid profiling and cardiovascular risk estimations based on the relations between the levels of long-chain omega-3 fatty acids for coronary heart disease and fatal ischemic heart disease [3-5]. The wide diversity in the diets (including fish intakes) and use of supplements or functional foods containing DHA+EPA in this free-living population provided a convenient spread of blood levels of omega-3 fatty acids and fatty acid ratios. The receiving and processing of these samples were approved by the Human Ethics Committee of the University of Guelph. Since the primary goal of this study was to evaluate the relationship between the levels (percentages) of total omega-3 fatty acids, DHA, and (EPA+DHA) in the biomarker (serum phospholipid) and specific fatty acid ratios (omega-6:omega-3, AA/EPA, AA/DHA, and AA/(EPA+DHA)), no restriction was placed on the subjects (including blood sampling being permitted in the fasted or postprandial state). Following collection of the blood samples by venipuncture into a Vacutainer tube, the serum was collected for processing and analyses via centrifugation [10,11].

### Fatty Acid Analyses of Serum Phospholipid

Fatty acid compositions of total serum phospholipid were determined based on previous studies [10,11]. Lipids were extracted from the serum samples according to the method of Folch et al.[12] and the serum phospholipids were separated from the neutral lipids by thin-layer chromatography [10,11]. The fatty acids methyl esters were prepared from the isolated phospholipid fraction by the method of Morrison and Smith [13] and were analyzed on a Varian 3400 gas-liquid chromatograph (Palo Alto, CA) with a 60-metre DB-23 capillary column (0.32 mm internal diameter).

### Statistical Analysis

The database was assessed for completeness and it was determined that a total of 2053 blood test analyses contained results for each of the bio-markers which were to be analyzed. The database was formatted in a Microsoft Excel™ (EXCEL) spreadsheet. A preliminary analysis using the EXCEL correlation coefficient function and trend-line analysis graphing function was performed on all of the data. Upon review of the resulting correlation coefficients for all comparisons, it was determined that statistically significant correlations could be ascertained for the dependent variables (x-axis) as Total Omega-3, DHA, and (EPA+DHA).

Independent variables (y-axis) for "yTotal Omega-6/Total Omega-3", "AA/EPA", "AA/DHA", and "AA/(EPA+DHA)" ratios were selected. Data for EPA, DHA, total Omega-3, total Omega-6, and AA were extracted from the original spreadsheet into a new spreadsheet for analytical purposes. The EPA and DHA levels were summed to calculate "EPA+DHA" values for each of the samples. Calculations were made for the "Total Omega-6/Total Omega-3", "AA/EPA", "AA/DHA", and "AA/(EPA+DHA)" ratio values for each sample. Calculations using the correlation function were made using linear, polynomial, log, exponential and power curve fits. In every case, the power curve provided the highest correlation coefficient and Power was therefore selected to model the data. A curve based on the Power function was plotted and the R^2 ^was recorded. This curve is used for graphical purposes only. The R^2 ^was used to compare between the different Y-values (i.e. AA/EPA ratio, n-6/n3 ratio, etc) so that a determination could be made as to which ratio "fit" the data best. The Power Function was not used in determining the cut-offs, these cut-offs are based on distribution calculations as described below [14].

The ninety-five percent (95%) confidence intervals [15] for the new independent variable cut-offs (selected fatty acid ratios) were established by analyzing the data which was above the known cut-off values for each dependent variable (fatty acid components as % of total fatty acids in serum phospholipid ) and their associated 'lower risk' status.

Calculations were validated by the University of Guelph's Department of Mathematics and Statistics.

## Results

The fatty acid data available from our very large sample (n = 2053 subjects) on the levels of total omega-3, DHA, and EPA+DHA as percentages of total fatty acids in serum phospholipid is considered one of the largest ever processed. Based on the estimated 'lower risk' categories for the risk of cardiovascular disease-related outcomes [3-5], the proportion of our population considered to be at potential 'risk' could be determined. Based on a 'risk' cut-off' of ≥ 7.2% for total omega-3 fatty acids and ≥ 4.5% for DHA [3,4] as % of fatty acids in serum phospholipid, 49.5% (1017 of 2053) and 53.9% (1106 of 2053) of our samples would be placed in the 'risk' category. In the case of EPA+DHA levels, where % values of ≥ 4.6 appears associated with a considerably lower risk of fatal ischemic heart disease [4,5], 34.0% (699 of 2053) could be classified in the 'risk' category.

**Figure 1:**
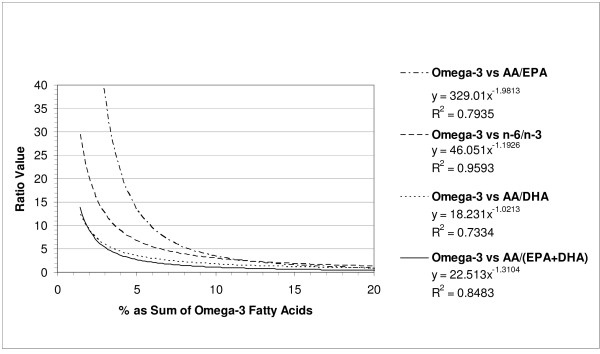
**Relationships between % of Total Fatty Acids in Serum Phospholipid as Omega-3 and Fatty Acid Ratios**.

Statistical analysis revealed very strong and significant inverse correlations between the sum of total omega-3 fatty acids in serum phospholipid and all four ratios (Figure 1) with the most potent correlation (R^2 ^= 0.96) being with the omega-6 fatty acids (sum): omega-3 fatty acids (sum) ratio followed (in order of diminishing potency) by the ratios of AA:(EPA+DHA), AA:EPA, and AA:DHA.

**Figure 2:**
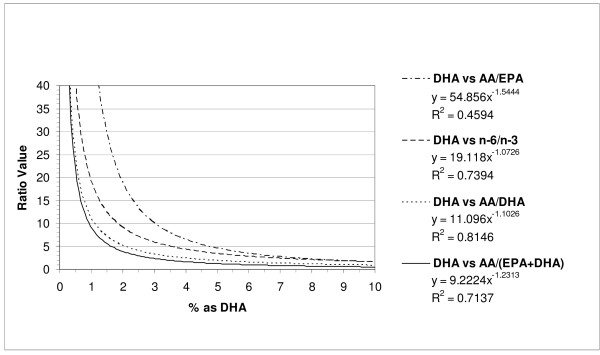
**Relationships between % of Total Fatty Acids in Serum Phospholipid as DHA and Fatty Acid Ratios**.

In the case of % of fatty acids as DHA in serum phospholipid (Figure 2), the most potent inverse correlation was found with the AA:DHA ratio (R^2 ^= 0.81) and the weakest correlation was with the AA:EPA ratio (R^2 ^= 0.46). The other two correlations with the omega-6:omega-3 ratio and the AA: (EPA+DHA) fell between these extremes with R^2 ^values of 0.74 and 0.71, respectively.

**Figure 3:**
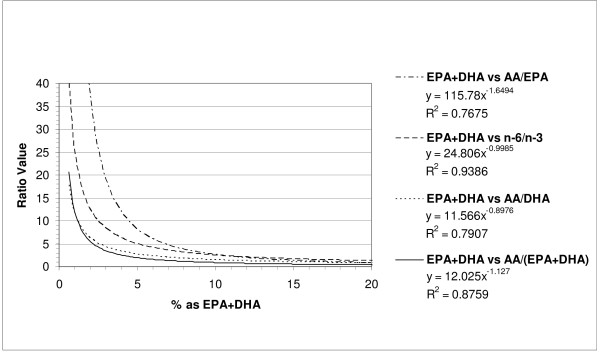
**Relationships between % of Total Fatty Acids in Serum Phospholipid as EPA+DHA and Fatty Acids Ratios**.

Correlational analyses for the % of total fatty acids in serum phospholipid (Figure 3) as EPA+DHA showed very strong inverse relations with all four ratios. The strongest relation was found with the omega-6:omega-3 ratio (R^2 ^= 0.94) followed closely by the AA: (EPA+DHA) ratio (R^2 ^= 0.88) and then the AA:DHA (R^2 ^= 0.79) and AA:EPA (R^2 ^= 0.77) ratios.

Table 1 gives the cut-off values for the four fatty acid ratios which would place 95% of the fatty acid levels in the 'low risk' category for coronary heart disease (total omega-3 ≥ 7.2 and DHA ≥ 4.5% of total fatty acids in serum phospholipid) and fatal ischemic heart disease (sum EPA+DHA ≥ 4.6%).

**Table 1:** Cut-off Values for the Fatty Acid Ratios which place the Levels of Total Omega-3 Fatty Acids, DHA, and EPA+DHA in the 'Lower Risk' Categories with 95% Confidence Level

	Cut-offs for Fatty Acid Ratios			
	
Fatty Acid Levels * (Lower Risk Category)	Omega-6:Omega-3	AA:EPA	AA:DHA	AA:(EPA+DHA)
Total Omega-3 (≥7.2)	<4.5	<5.0	<1.8	<1.4
DHA (≥4.5)	<3.0	<1.1	<1.5	<0.45
EPA+DHA (≥4.6)	<5.8	<9.2	<2.5	<2.1

* The 'lower risk' categories for total omega-3, DHA, and EPA+DHA levels (as % of total fatty acids in serum phospholipid) are for coronary heart disease in the case of total omega-3 and DHA [3,4] and for fatal ischemic heart disease in the case of EPA+DHA [4,5].

For each of the latter three parameters, the lowest numerical cut-off ratios were for AA:(EPA+DHA) showing cut-offs of <1.4, <0.45, and <2.1, respectively. The highest numerical cut-off ratios for total omega-3, DHA, and EPA+DHA were AA:EPA (<5.0), omega-6:omega-3 (<3.0), and AA:EPA (<9.2), respectively.

## Discussion

The present results indicate that very strong and highly significant inverse relationships exist for the levels of total omega-3 fatty acids, DHA, and EPA+DHA (sum) as a % of total fatty acids in serum phospholipid (Figures 1, 2 and 3). These results are of interest since the levels of these fatty acids have been correlated with the risk for coronary heart disease and fatal ischemic heart disease [3-5]. Furthermore, various groups have reported on either these fatty acid levels or ratios such as the omega-6:omega-3, AA:EPA, and EPA:DHA ratios but not both simultaneously in chronic disease risk assessment [3-5,7-9]. Our present study provides some guidance as to what appropriate ratio cut-offs may be considered for cardiovascular disease risk assessment (Table 1). Interestingly, the most potent relationships (inverse) for any fatty acid parameter and ratio was for total omega-3 fatty acids and the omega-6:omega-3 ratio (R^2 ^= 0.96). In this case, a ratio of < 4.5 would place 95% of the subjects in a 'lower risk' category for coronary heart disease based on an omega-3 (summed) risk cut-off of ≥ 7.2% [3,4]. As might be expected, the AA:DHA ratio exhibited the strongest inverse relation to the DHA levels. The omega-6:omega-3 ratio also showed the strongest inverse relation to the EPA+DHA levels in serum phospholipid (R^2 ^= 0.94) and a cut-off ratio of <5.8 would be expected to place 95% of the subjects in a 'lower risk' category for fatal ischemic heart disease based on ≥4.6% of the total fatty acids being present as EPA+DHA [4,5].

The major fatty acids contributing to the total omega-3 fatty acids in human serum phospholipid are EPA+DHA along with docosapentaenoic acid with alpha-linolenic acid being a very minor component despite its abundance in dietary plant oils with AA plus LA (linoleic acid, 18:2 n-6) being the major contributors to the total omega-6 fatty acids [16,17]. Based on typical baseline fatty acid ratios [16] in serum phospholipid for groups of North American males (omega-6: omega-3 = 8.1, AA:EPA = 16.2, AA:DHA = 4.2, and AA:(EPA+DHA) = 3.3)), none of these average ratios fall in the lower risk categories as defined in Table 1. Japanese males [17] with much lower corresponding ratios of 2.3, 1.7, 0.9, and 0.6, respectively, would fall in the 'lower risk' categories for coronary heart disease based on total omega-3 (≥7.2) and fatal ischemic heart disease (EPA+DHA ≥4.6) based on Table 1. Their chronic daily intake of EPA+DHA, mainly from fish/seafood, was estimated to average 1500 mg/day [17]. Interestingly, daily supplementation of North American males with 630 mg EPA plus 640 mg DHA for 21 days lowered their ratios into or towards the 'lower risk' categories with newly-modified ratios of 3.8, 5.1, 2.1, and 1.5, respectively [16].

It is of interest to note that the omega-3 index, a measure of the % of total fatty acids in the red blood cell represented by the sum of EPA + DHA, has been suggested as a risk factor for death from coronary heart disease [18]. Also, a very strong correlation between the omega-3 index and the % of total fatty acids in plasma phospholipid as EPA+DHA (r = 0.91) was reported [18]. The cut-off value referred to herein (EPA+DHA in serum/plasma phospholipid) based on the literature for a reduced risk of fatal ischemic heart disease [4,5] of ≥ 4.6% can be compared to ≥8.0% for the omega-3 index [18]. Interestingly, the ≥8.0% level gives a value of ≥ 4.7% for EPA+DHA if the former is extrapolated to plasma phospholipid based on the correlation equation provided [18].

It needs to be emphasized that the relations between the fatty acid levels and the ratios presented herein are considered independent of the health or clinical condition of interest. Specific information obtained on the majority (70%) of the subjects in our population providing blood samples (numbering 2053) indicated that 54% were males and 46% were females. Of these, the average age for both genders was 54.7 years with the average for males and females being 55.2 and 54.2 years, respectively.

The estimated 'risk' fatty acid ratios and cut-offs as presented herein were directed to cardiovascular disease and fatal ischemic heart disease and could be of value if/when complete profiles are not available. Thus, they would not, of course, apply to depression, neuroticism, and other chronic conditions where ratios have been of interest [7,8]. It is also emphasized that the biomarker for the present study is serum phospholipid such that plasma phospholipid but not whole blood or red blood cell data could be substituted.

## Conclusions

In summary, our study showed robust inverse curvilinear relationships between the summed omega-3 fatty acids in serum phospholipid (biomarker) as well as the (EPA+DHA) levels and the omega-6/omega-3 and the AA/(EPA+DHA) ratios based on fatty acid analyses of 2053 human blood samples. Omega-6/omega-3 ratios <4.5 were estimated to support a 'lower risk' category with 95% confidence for the risk of coronary heart disease while AA/(EPA+DHA) ratios <2.1 would be expected to provide a corresponding lower risk category for fatal ischemic heart disease.

## List of Abbreviations

AA: Arachidonic acid, 20:4 n-6; DHA: Docosahexaenoic acid, 22:6 n-3; EPA: Eicosapentaenoic acid, 20:5 n-3; LA: Linoleic acid, 18:2 n-6

## Competing interests

B.J. Holub, M. Wlodek, and W. Rowe all hold ownership shares in Nutrasource Diagnostics Inc. This company provides omega-3 fatty acid blood testing services for health care groups and their clients in North America. J. Piekarski holds an ownership share in Lipid Analytical Laboratories Inc.

## Authors' contributions

BJH was responsible for the writing of this manuscript including interpretation of the data. MW contributed to the statistical analyses of the data with advice from the Department of Mathematics and Statistics, University of Guelph. WR served as project coordinator. JP performed all the serum phospholipid fatty acid analyses on the blood samples. All authors have read and approved the final manuscript.
